# Long-term radiographic and clinical-functional outcomes of isolated, displaced, closed talar neck and body fractures treated by ORIF: the timing of surgical management

**DOI:** 10.1186/s12891-019-2738-2

**Published:** 2019-08-07

**Authors:** Carlo Biz, Nicolò Golin, Michele De Cicco, Nicola Maschio, Ilaria Fantoni, Antonio Frizziero, Elisa Belluzzi, Pietro Ruggieri

**Affiliations:** 1 Orthopaedic and Traumatology Clinic, Department of Surgery, Oncology and Gastroenterology DiSCOG, University of Padova, via Giustiniani 2, 35128 Padova, Italy; 2 Department of Physical and Rehabilitation Medicine, University of Padova, Padova, Italy; 3 Musculoskeletal Pathology and Oncology Laboratory, Department of Surgery, Oncology and Gastroenterology DiSCOG, University of Padova, Padova, Italy

**Keywords:** Talar fractures, Talar neck fractures, Talar body fractures, Talus, ORIF, Screw fixation

## Abstract

**Background:**

The main purpose of this retrospective case series study was to evaluate long-term radiographic and clinical outcomes of a consecutive series of patients diagnosed with isolated, displaced, closed talar neck or body fractures treated by open reduction and internal fixation (ORIF). Secondly, the aim was to verify the influence of the location of talar fractures on the outcomes, the prognostic value of the Hawkins sign, whether operative delays promote avascular necrosis (AVN) and if the fractures require emergent surgical management.

**Methods:**

From January 2007 to December 2012, at our institution, 31 patients underwent ORIF through the use of screws. On the basis of Inokuchi criteria, the injuries were divided between neck and body fractures, which were classified according to Hawkins and Sneppen, respectively. The patients included were divided into two groups in relation to fracture location and complexity. Radiographic assessment focused on reduction quality, bone healing, the Hawkins sign and post-traumatic arthritis (PTA) development. For the clinical evaluation, clinical-functional scores (AOFAS Ankle-Hindfoot Score; MFS; FFI-17; SF-36) and VAS were determined, and statistical analysis was performed.

**Results:**

27 patients, 19 males and 8 females, mean age 38.3 years, were included with an average follow-up period of 83.2 months (range 49–119). There were 9 neck and 19 body fractures; their reduction was anatomical or nearly anatomical in 22 cases, and all reached radiographic consolidation after a mean period of 3.4 months (range 1.7–7). The Hawkins sign was observed in 9 cases, in which necrosis did not develop. With a 0–11 day surgical timing interval, more than 60% of the patients obtained good or fair results with different scores, while 18 (66.7%) were completely satisfied (VAS: 9–10). The early complications included malunions (21.4%) and wound problems (25%); the late complications involved AVN (25%) and PTA (78.6%).

**Conclusions:**

Despite a high rate of long-term complications, satisfactory clinical results were achieved. Talar fracture location did not influence the outcomes, the Hawkins sign was confirmed as a positive prognostic factor, and operation timing did not influence AVN development. Hence, these injuries do not require emergent surgical management by ORIF*.*

## Background

Talar fractures are rare, accounting for less than 1% of all fractures in the human body and estimated to comprise between 3 and 6% of all foot fractures [1–3]. However, they are described as being among the most challenging injuries to manage, even for experienced orthopaedic and trauma surgeons due to their various locations and patterns, the unique anatomical shapes and the vascular anatomy of the talus bone, as well as the choice of conservative or aggressive treatment and relative timing, surgical approaches and internal fixation hardware [3–6]. The mechanism of injury generally consists of the application of a sudden dorsiflexion force on a fully plantar-flexed foot, thereby imparting a compressive force through the talar head, or a hyperdorsiflexion, resulting in compression of the talar head against the anterior tibial edge. When, during trauma, the artery of the tarsal canal with its large extra- and intraosseous anastomotic vascular network to the sinus tarsi artery [7–9] is disrupted, the talus might sustain impaired vascularity.

Despite the evolution of diagnostic techniques and the improvement of various approaches described for safe dissection and the safety of different surgical procedures achieved in recent years [5, 6], complications remain extremely frequent. In particular, the avascular necrosis (AVN) rate in talar neck and body fractures is reported between 12 to 53% [2, 10, 11]. As in the past, several authors have considered fracture displacement and delayed surgery the main risk factors for this daunting complication; immediate ORIF has been historically recommended to decrease its incidence [12]. On the contrary, the most recent literature has shown that there is no correlation between time of surgical fixation and development of osteonecrosis [5, 11]. Further, associated swelling and soft tissue damage, which significantly increase early soft tissue sequelae, should be taken into account before definitive surgery [13, 14]. Different reports published on this topic during the years - mostly retrospective, often with follow-up periods too short to discuss the outcomes critically and only a few specifically focused on the isolated injuries - have also contributed to the treatment dilemma for talus fracture-dislocations in many facilities [1–3, 11, 15–19].

Hence, the primary aim of our retrospective, non-randomized case series study was to specifically evaluate the long-term radiographic and clinical outcomes of a consecutive series of patients diagnosed with isolated, displaced, closed talar neck or body fractures treated at our institution by ORIF. Secondly, based on the data found, we aimed to verify the influence of talar fracture location on the outcomes, the prognostic value of the well-known Hawkins sign, whether the operative delays promote AVN development and if the fractures effectively do not require emergent surgical management.

## Methods

### Patients

In this retrospective case series study, we examined clinical and radiographic data from a consecutive series of Caucasian patients diagnosed with isolated, displaced, closed talar fractures. At our Level I healthcare trauma centre, between January 2007 and December 2012, these patients underwent ORIF using 3.5 mm cortical, cannulated screws or 4.0 mm lag screws (DepuySynthes, Umkirch, Germany). All subjects participating in this study received a thorough explanation of the risks and benefits of inclusion and gave their oral and written informed consent to publish the data. This study was approved by the Institutional Ethics Committee (N° 4065/AO/17) and performed in accordance with the ethical standards of the 1964 Declaration of Helsinki as revised in 2000 and those of Good Clinical Practice.

### Inclusion and exclusion criteria

The inclusion criteria were the diagnosis of a closed, isolated, displaced talar neck or body fracture with 2 or more millimetres displacement, subsequently treated by ORIF. All patients considered in this study had to be between 18 and 85 years of age and give their informed consent to participate. Specific patient exclusion criteria included undisplaced fractures or involvement of both the neck and the body, open fractures, talar head and peripheral fractures including posterior process, osteochondral fractures, primary arthrodesis or amputation, a history of severe neurological deficit, previous foot surgery or trauma, diagnosis of rheumathological diseases or psoriatic arthritis, foot neuropathy, severe vascular insufficiency and alcohol or drug abuse.

### Surgical technique

All operative procedures were performed by one of our trauma team surgeons, including the senior author (C.B.), with the help of two different residents of our institution. In all operations, plexus anaesthesia was performed consisting in a regional block, which involved both sciatic and femoral nerves (bi-block), while sedation was used when necessary. Prophylactic cefazolin (2 g) was administered and continued 24 h after surgery. Postoperative antithrombotic therapy (Natrium Enoxaparin) was given until weight bearing. The patient was placed supine on a radiolucent operating table, with the foot elevated on an appropriate support; a thigh tourniquet was always applied, even if it was inflated only to control bleeding. Although the current surgical fixation strategy of talar fractures provides for the use of both the anteromedial (AM) and anterolateral (AL) approaches to optimize visualization of the entire talar neck [15, 20–24], a single AM or AL approach was used to prevent soft-tissue complications, through an incision of approximately 10 cm in length made depending on the location of the fracture. Subsequently, the extensor retinaculum was sharply incised, and the tendons retracted to improve visualisation. Also in the case of complex neck and body fractures, a single approach was used extending the standard incision by a couple of centimetres in a curved manner, enough to spread the skin without tension: more proximally with respect to the anteromedial aspect of the medial malleolus and more distally to the medial cuneiform for the AM approach; or more proximally with respect to the Chaput tubercle and more distally toward the base of the fourth metatarsal bone for the AL approach. In this way, it was possible to perform a wider L- or T-shaped incision of the ankle joint capsule, which was crucial to expose the fractures of the neck or body of the talus. After cleaning the fracture site to make it visible, but avoiding dissection around the talus to prevent additional devascularization of the tenuous blood supply, manual anatomical reduction by Weber pointed reduction clamps was performed. The same procedure was used for talar body fractures through the use of an AM approach, proximally extending the incision [18], but avoiding the medial malleolar osteotomy and possible damage of the deltoid ligament, which is an important blood supplier to the talar body [25]. In fact, for this case series, no combined or associated malleolar osteotomies were carried out, nor was bone grafting employed.

Subsequently, the provisional fixation of fragments was achieved using temporary Kirschner wires under radiographic guidance. Having obtained satisfactory reduction as seen with the radiographic intensifier, definitive fixation of the main bone fragments was carried out using two or more 3.5 mm titanium cannulated screws or 4.0 mm lag screws, placed anteriorly to posteriorly. Fractures complicated by severe comminution necessitated 3.5 mm cortical screws in order to avoid shortening, translation or angulation of the fragments. No definitive K-wires were left in place, nor were mini-plates used in this series. A non-weight-bearing cast in neutral ankle alignment was applied and maintained for a period of 4 weeks, and the patients were kept non-weight-bearing on the operated limb using two crutches for 8 weeks. Passive and active ankle ROM exercises were allowed after plaster removal. Progressive weight-bearing combined with physiotherapy was suggested after this period.

### Patient assessment

Data collection was retrospectively performed at our institution by two external and independent investigators, the junior authors (N.G. and M.D.C, respectively), not involved in the patients’ treatment. Patients’ characteristics (gender, age at trauma, BMI, comorbidities, ASA class to globally estimate surgical risk [26], smoking habits), trauma characteristics (affected side, mechanism, concomitant injuries), treatment characteristics (surgical delay, duration of surgery, hospitalization) and post-operative characteristics (non-weight bearing, early and late complications, the Hawkins sign) were collected from the electronic database of the hospital. Furthermore, the clinical and radiological analyses were carried out respectively by two independent researchers who were not directly involved in the patients’ surgical treatment (N.M. and I.F.). Finally, the patients included in the investigation were divided into two initial groups according to the fracture location [27]:

1. *The talar neck fracture group:* patients with any fracture line lying between the talar head and body (lateral process);
2. *The talar body fracture group:* patients with any fracture line at/or posterior to the lateral tubercle of the talus).

The patients were further divided into two groups according to fracture complexity, depending on fragment displacement or comminution [28, 29]:

1. *The simplex fracture group:* patients with fractures Hawkins type II; Sneppen type II, III and V;
2. *The complex fracture group:* patients with fractures Hawkins type III and IV; Sneppen type VI.

### Radiographic outcome measures

Radiographic data were obtained by reading the radiographic computerized images available in the computer system of our institute. The radiographic evaluation comprised the analysis of conventional radiographs, including anteroposterior (A/P), lateral (L/L) and oblique ankle views in the preoperative, postoperative and follow-up periods and preoperative CT scans, when performed. All radiological evaluations were performed with the Medstation program (the X-ray data base of our hospital).

This software, in association with a Diagnostic LCD CORONIS 5MP display (produced by Barco, Rome, Italy) viewing monitor to analyse the fractures and their outcomes, allows the retrieval of electronically computer-assisted measurements from radiographs, even for short linear distances (2 mm) or reduced angular values (5°) combining high magnification with excellent resolution. Hence, for this study it was possible to assess small angular values by tracing the reference lines even if the vertex of the angle itself fell outside the radiographic image using the function called “angle measurement between lines”.

On the basis of the preoperative x-rays, the fractures were classified in accordance with Inokuchi [27], who defined talar body fractures as having a fracture line posterior to the lateral process of the talus on the inferior face of the talus, while neck fractures are those located in front of this process. Talar neck fractures were then classified in accordance with Hawkins, as modified by Canale and Kelly [30], while talar body fractures were classified in accordance with Sneppen [29].

The following radiographic examination of the post-operative radiographs permitted us to:

- evaluate the quality of the reduction immediately after surgery. Any offset of more than 2 mm or neck angulation of more than 5° between the fragments was labelled a poor reduction [4];
- assess the complete bridging bone/callus formation and the absence of radiolucent lines, used as criteria to define bone healing and union at different follow-ups;
- verify the Hawkins sign appearance (only on the A/P X-ray) at 6–8 weeks after injury, which resembles a subchondral atrophy in the talus dome, indicating that the talus is well vascularized. On the contrary, its absence at this time suggests the presence of osteonecrosis;
- determine the development of post-traumatic arthritis (PTA) and differentiate between necrosis without collapse (sclerosis with and without subchondral cysts) and necrosis with collapse of the talar dome at the last follow-up.

### Functional outcome measures

At the time of this study, a phone contact was attempted for all patients who met inclusion criteria, and a follow-up appointment was fixed. Patients who returned were examined, and clinical results were measured with validated questionnaires. To quantify pain and functional disability, the American Orthopaedic Foot and Ankle Society (AOFAS) Ankle-Hindfoot score [31], the Maryland Foot Score (MFS) [32] and the 17-Foot Functional Index (FFI-17) [33] were used. The AOFAS questionnaire includes 9 questions related to pain (1 question; 40 points), function (7 questions; 50 points) and alignment (10 questions; 10 points); a score of 90–100 is considered an excellent result; 75–89 as good; 50–74 as fair and less than 49 points is considered a failure or a poor outcome. The MFS is a scoring system conceptually analogous to AOFAS score, but points are differently distributed (45 for pain, 55 for functional limitation); they indicate excellent results if the score is between 90 to 100, good for a score of 75 to 89, fair for a score of 50 to 74 and poor if the score is < 50. The FFI-17 measures the persistence of pain, disability and restriction of activity, with 17 number-rating scales from 0 to 10. The maximum score is 100, which indicates complete disability. All patients were also asked to complete the Short Form 36 (SF-36) [34], which is a validated questionnaire widely used for different pathologies to measure the patient-reported quality of life. It consists of 36 questions, representing 8 health domains that are combined into physical (PCS) and mental component summaries (MCS), using the US population as reference. For this analysis, both summary scores were used. Further, a 0–10 visual analogue scale (VAS) was used to quantify patient satisfaction of the results, where 0 means maximum dissatisfaction and 10 full satisfaction. The patients were also queried regarding shoe-related problems, as well as work and sports activities at the age of the trauma and their resumption. In particular, hindfoot inversion and eversion mobility was evaluated by dividing patients into four categories of stiffness: absent, mild, moderate and severe. Finally, any complications were recorded.

### Statistical analysis

Statistical analyses were performed by an independent statistician from the Department of Statistics at our University, blinded to the type of injuries. Analysis of data was performed using SAS 9.2 (SAS Institute Inc., Cary, NC, USA) for Windows. Continuous data were checked for a normal distribution with the Shapiro-Wilk test and expressed with average and standard deviation or median and minimal-maximal value. Results were compared among groups with different fracture localization and different injury severity. Fisher’s exact tests for categorical variables and analysis of variance or the Wilcoxon two-sample test for continuous variables were used. Spearman’s rank correlation coefficient was used to evaluate the influence of the surgical delay on AVN development and clinical outcomes. The correlation between trauma and patient characteristics and the onset of complications was tested with univariate logistic regression sequelae (a patient with more than one complication was considered only once); the odds-ratio and its 95% confidence interval were calculated. A *p* value of < 0.05 was taken as the threshold of statistical significance.

## Results

### Patient data

During a six-year period, 31 patients with 33 fractures were treated at our institution. We could not evaluate 4 patients (5 fractures), as one refused to participate, and a follow-up address could not be retrieved for 3 people. Hence, 27 patients with 28 fractures (one patient presented bilateral fractures) were treated at our institution. The patients’ details are summarized in Table 1. There were 19 men (1 bilateral case for a total of 20 fractures, 71.4%) and 8 women (29.6%). Overall, mean age at the time of injury was 38.3 years old (range 18–81). The average follow-up period was 83.2 months (range 49–119), i.e., almost 7 years. The only trauma mechanisms reported were a fall from a height in 13 patients (48.1%) and road accidents in 14 (51.9%); for the latter, causes of fractures were car accidents in 8 cases (28.6%), motorcycle accidents in 5 cases (17.9%) and a pedestrian accident in 1 case (3.6%). Patient comorbidities and risk factors were recorded as well: mean BMI was 23.8 kg/m^2^ (± 3.7), and 1 subject was obese (BMI > 30); active smokers were 8 (29.6%); 4 patients (14.8%) reported hypertension, 2 (7.4%) diabetes and 2 (7.4%) heart disease (previous myocardial infarction, arrhythmias, valvular heart disease) or vascular disease. According to the ASA (American Society of Anaesthesiologists) classification of globally estimate surgical risk, there were 19 patients ASA 1 (70.4%), 7 ASA 2 (25.9%) and 1 ASA 3 (3.7%). During the six-year period, 20 talus osteosyntheses on the right foot and 8 on the left were performed (on average 4.7 operations every year), through a single anteromedial approach in 16 cases (57.1%) and an anterolateral approach in 12 cases (42.9%). Time between trauma and surgery ranged from 0 to 11 days with a median time of 2 days. The mean duration of surgery was 105 min (range 70–150).

**Table 1:** Demographic data, fracture types, follow-up period, complications and clinical-functional scores of our case series

Patient n°	Gender, Age (years)	Fracture Types	Post-operative Reduction	Follow-Up (months)	Early Complications	Late Complications	AOFAS	MFS	FFI-17 (%)	VAS	SF-36	
											PCS	MCS
1	M, 26	S III	Anatomical	119		PTA TNJ	95	97	17.6	10	81.4	77.9
2	M,18	S V	Anatomical	117		PTA TNJ	97	99	7.6	10	84.9	58.8
3	F, 41	H II	Nearly Anatomical	116	Wound dehiscence	PTA TTJ	93	90	9.4	10	77.4	73.1
4	M, 36	S VI	Poor	111	Cutaneous necrosis, malunion	PTA STJ, TTJ, TNJ; AVN	59	53	68.2	8	44.3	38.9
5	F, 30	H II	Poor	108	Malunion	PTA STJ, TTJ, TNJ	41	57	55.3	9	66.9	71.6
6	M, 44	H II	Nearly Anatomical	107		PTA STJ	66	82	51.2	10	59.5	32.7
7	M, 23	S III	Nearly Anatomical	103		PTA STJ	73	77	23.7	9	69.4	70.8
8	M, 23	S II	Nearly Anatomical	101			93	88	25.3	7	78.4	46.2
9	M, 27	S II	Anatomical	99			90	93	17.5	8	80.9	88.7
10	F, 28	H II	Nearly Anatomical	95		PTA STJ	62	67	14.7	8	55.6	68.7
11	M, 21	S V	Anatomical	91		PTA TNJ	90	91	19.4	9	82.8	74.2
12	M, 42	S VI	Poor	90	Malunion	PTA STJ, TTJ, TNJ, AVN	55	69	53.5	10	43.6	63.3
13	M, 40	H III	Nearly Anatomical	88		PTA STJ	81	78	21.2	9	76.7	79.1
14	M, 24	S V	Anatomical	85			85	96	18.8	9	71	71.2
15	F, 32	S V	Anatomical	82		PTA TTJ	80	84	22.3	9	73.7	77.4
16	M, 39	H II	Nearly Anatomical	79	Wound dehiscence	PTA STJ	74	78	30.1	8	69.2	73.2
17	M, 25	H II	Anatomical	75			90	97	7.6	10	83.8	86.8
18	M, 43	S II	Nearly Anatomical	71		PTA TTJ	90	98	14.7	10	85.8	72.6
19	F, 26	S VI	Nearly Anatomical	69	Wound dehiscence,	PTA STJ, AVN	84	80	12.3	9	75.1	78.2
20	M, 63	S II	Anatomical	67	Wound dehiscence	AVN	83	84	31.2	10	74.4	80.3
21	F, 40	S V	Anatomical	66			85	92	14.1	8	78.8	75.2
22 (right)	M, 81	S II	Poor	63	Malunion	PTA STJ, TNJ	70	82	26.5	10	70.8	76.1
22 (left)	M, 81	S III	Nearly Anatomical	61		PTA STJ	75	84	29.4	10	70.8	76.1
23	M, 23	S II	Anatomical	57		PTA TNJ	80	91	40.5	9	78.7	85.3
24	F, 71	H III	Poor	55	Cutaneous necrosis, malunion	PTA STJ, TTJ, TNJ, AVN	25	22	84.7	8	55.8	73.3
25	M, 62	S VI	Poor	54	Malunion	PTA STJ, TTJ, AVN	68	63	44.7	10	72.7	74.7
26	F, 41	H IV	Nearly Anatomical	51	Wound infection	PTA STJ, TTJ, AVN	66	65	54.7	6	41.6	40.9
27	M, 21	S VI	Anatomical	49		PTA TTJ	80	65	30.0	10	71.7	77.4

*F* female, *M* male, *H* Hawkins, *S* Sneppen, *AVN* avascular necrosis, *AOFAS* American Orthopaedic Foot and Ankle Society Ankle-Hindfoot Score, *MFS* Maryland Foot Score, *FFI-17* Foot Function Index; *VAS* Visual Analogue Scale (for satisfaction), *SF-36* Short Form-36 (*PCS* Physical Component Summary, *MCS* Mental Component Summary), *PTA* post traumatic arthritis, *STJ* subtalar joint, *TTJ* tibiotalar joint, *TNJ* talonavicular joint

### Radiographic outcomes

**Fig. 1:**
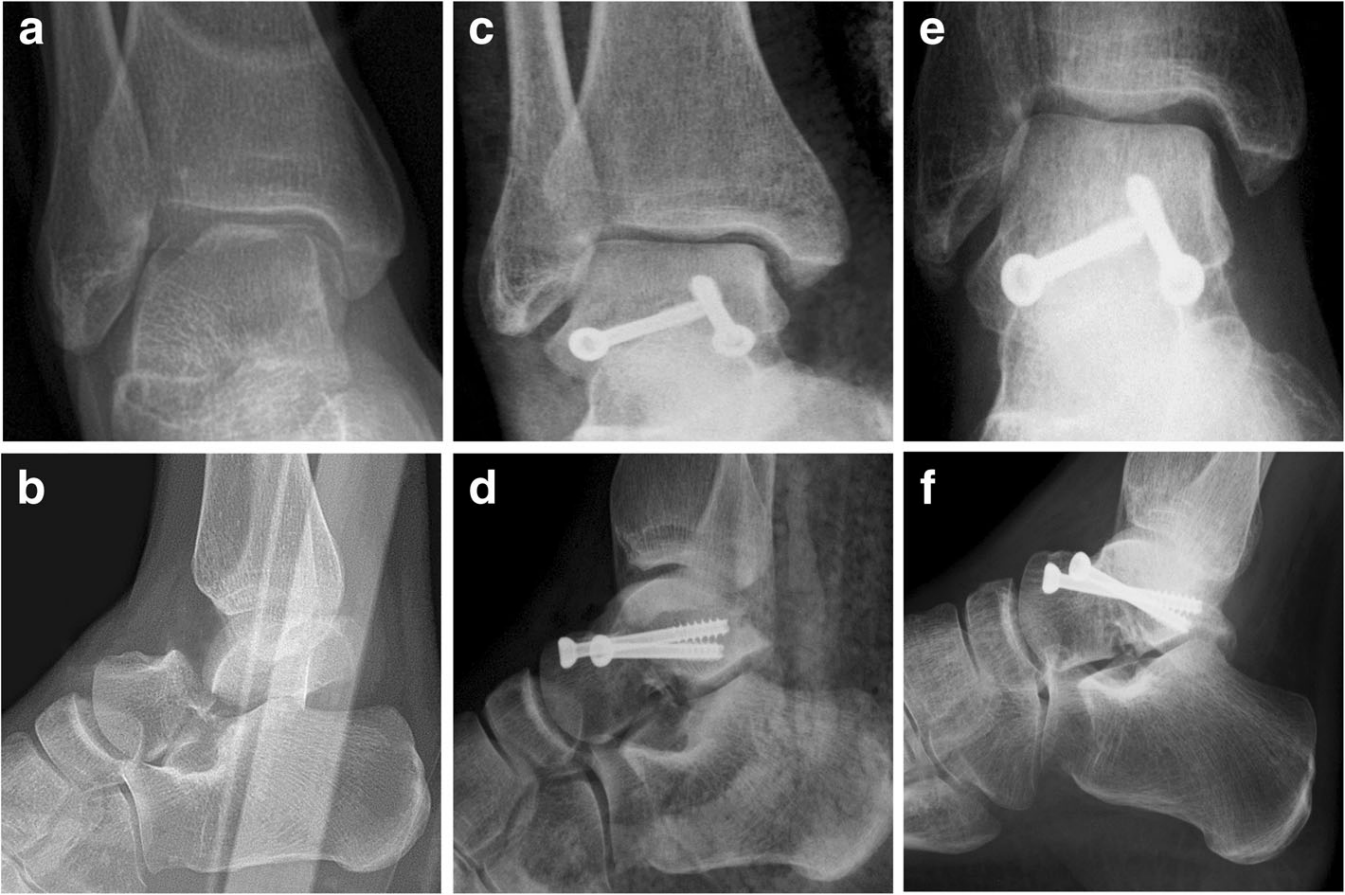
Case 1: a 40-year-old woman with a Hawkins type III fracture presenting signs of PTA and AVN, but without collapse of talar dome at last follow-up. Antero-posterior and lateral radiographic images at preoperative period (**a**-**b**), immediate postoperative period (**c**-**d**), 88-month follow-up (**e**-**f**)

**Fig. 2:**
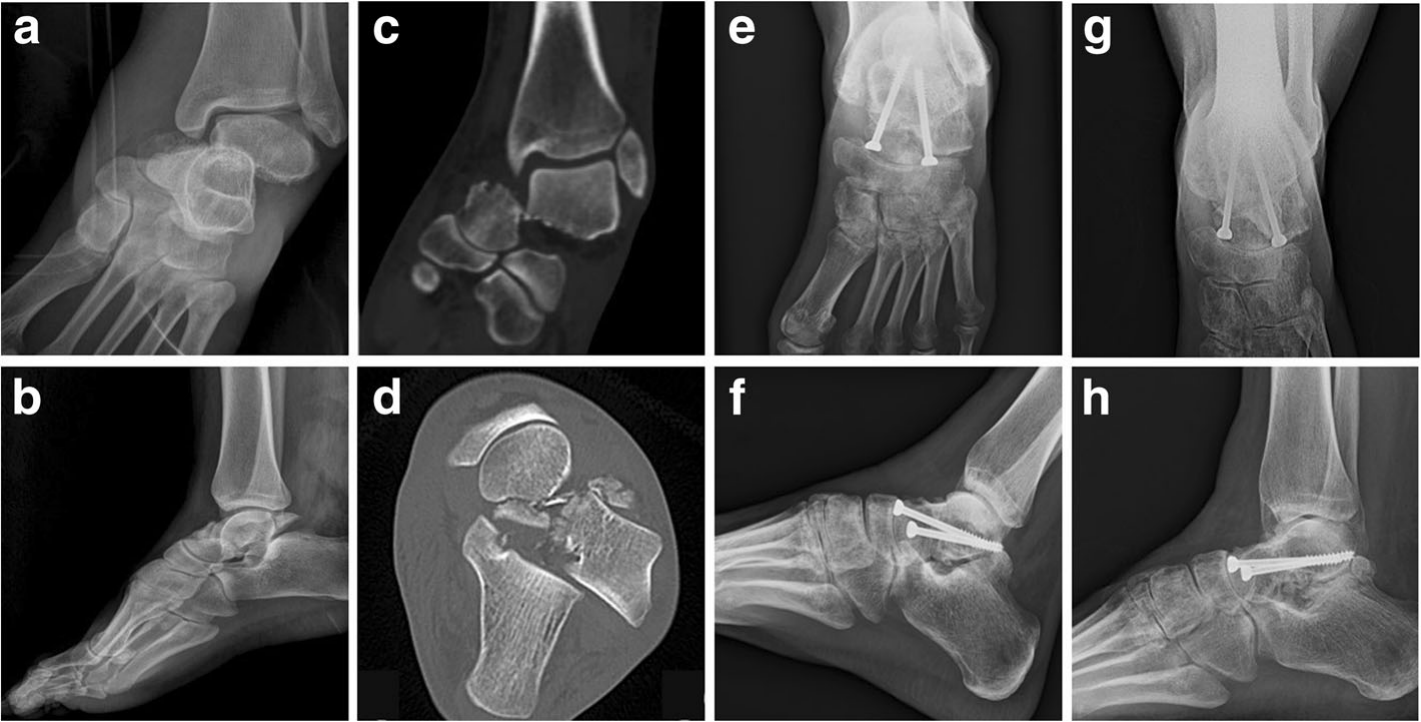
Case 2: a 41-year-old woman with a Hawkins type IV fracture presenting signs of union despite a disastrous initial condition. Antero-posterior and lateral radiographic images at preoperative period (**a**-**b**), coronal and axial CT images at preoperative period (**c**-**d**), antero-posterior and lateral radiographic images at immediate postoperative period (**e**-**f**), antero-posterior and lateral radiographic images at 51-month follow-up (**g**-**h**)

Preoperative radiological images including 8 ankle CT scans were analysed, and the fractures were classified and sub-classified using the three classification systems described. Based on Inokuchi criteria, there were 9 neck fractures and 19 body fractures. Among the neck fractures, according to Hawkins, 6 (21.4%) were type II fractures, 2 (7.1%) were type III (Fig.1), and 1 (3,6%) was a type IV fracture (Fig.2); while among the body fractures, according to Sneppen, there were 6 (21.4%) type II fractures, 3 (10.7%) type III, 5 (17.9%) type V and 5 (17.9%) type VI (Table 1). Hence, the *simplex fracture group* consisted of 20 fractures (71.4%), while the *complex fracture group* included 8 fractures (28.6%). In 22 cases (78.6%), the reduction resulted anatomical or nearly anatomical, while in 6 cases (21.4%), a poor reduction was observed (2 cases of the *neck fracture group* and 4 in the *body group*). All fractures treated achieved radiological consolidation after a mean period of 3.4 months (range 1.7–7) without non-union cases. Subsequent corrective osteotomies or hindfoot arthrodeses were not recorded at the final follow-up. The Hawkins sign was observed in 9 fractures (32.1%) with no patient suffering necrosis. Among 19 fractures with negative signs, there were 7 (36.8%) that developed necrosis, without however presenting collapse of the talus (sclerosis with and without subchondral cysts) at the last follow-up; while the other 12 fractures (63.2%) did not develop osteonecrosis.

### Clinical outcomes

**Fig. 3:**
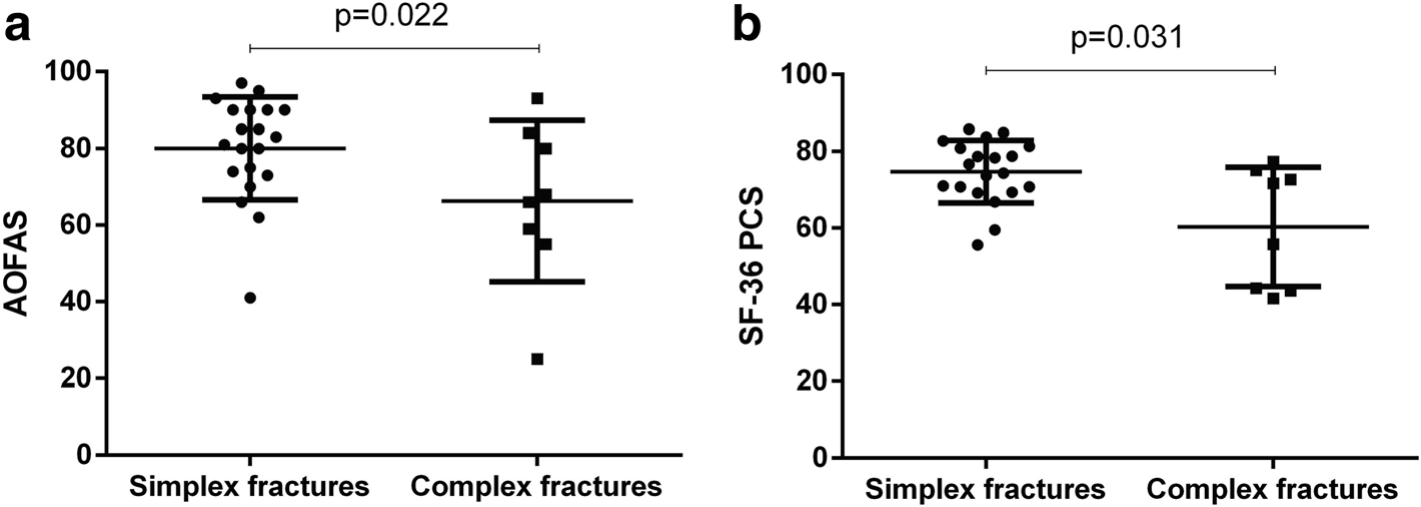
Frequency distribution (Wilcoxon rank sum test) of **a** AOFAS (*p* = 0.022) and **b** SF-36 PCS (*p* = 0.031) scores according to different fracture patterns (simplex and complex fractures)

**Fig. 4:**
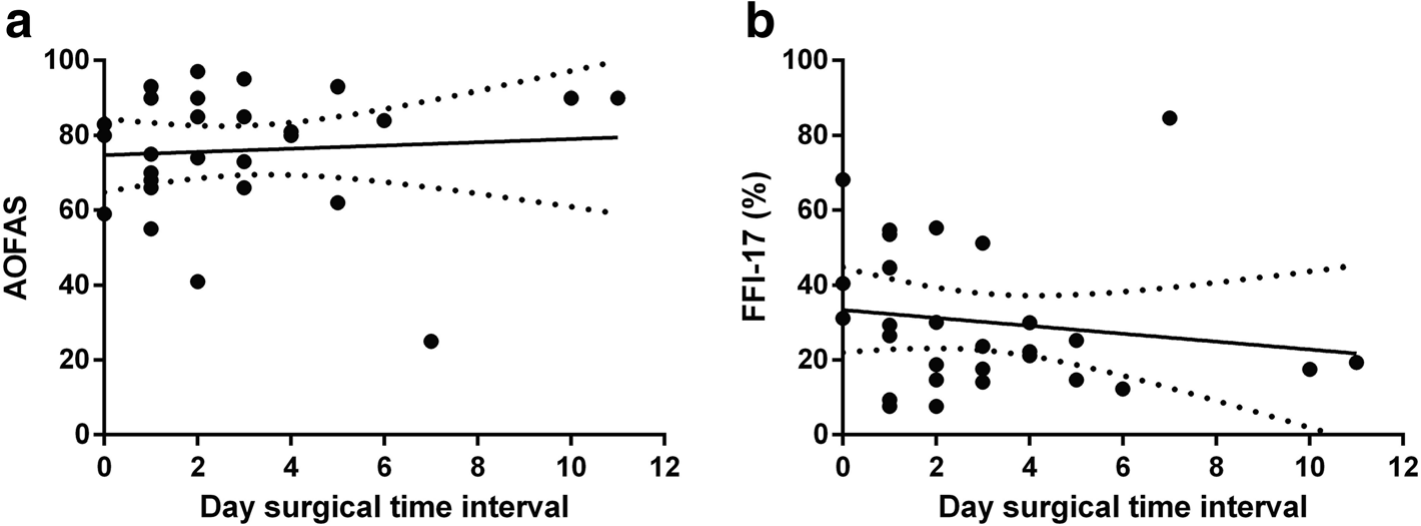
Correlation (Spearman’s rank correlation coefficient) between interval of trauma until ORIF in days and **a** AOFAS (*p* > 0.05, *p* = 0.811) and **b** FFI-17 (*p* > 0.05, *p* = 0.488) scores

In our cohort, the AOFAS scale measured excellent results (90–100 points) in 8 cases (28.6%), good results (75–89 points) in 9 cases (32.1%), fair results (50–74 points) in 9 cases (32.1%), while 2 (7.1%) fractures were graded as failures (< 50 points). Similarly, the MFS scale showed excellent results (90–100 points) in 10 cases (35.7%), good results (75–89 points) in 10 (35.7%), fair results (50–74 points) in 7 (25%), and there was 1 (3.6%) failure (< 50 points). With the FFI-17 questionnaire, 3 cases (10.7%) obtained optimal scores, lower than 10; in 14 cases (50%), the scores were between 10 and 30; in 5 cases (17.9%), between 30 and 50; and in 6 (21.4%), scores were higher than 50. According to the SF-36, the mean value of the physical component summary was 70.6 (range 41.6–85.8), while the mean value of the mental one was 70.1 (range 32.7–88.7). As for VAS, 20 patients (74.1%) were completely satisfied with a score of 9–10 (Table 1). Statistical analysis with pair comparison showed significant differences between the clinical-functional results of the *simplex fracture group* and the *complex fracture group* measured with the different scores (Fig.3), while the *neck fracture group* compared to the *body group* did not show statistically significant differences. With regard to VAS, no significant differences were observed between the *simplex fracture group* and the *complex fracture group* nor between the *neck fracture group* and the *body fracture group*. In our sample, 16 patients (59.3%) had practiced sports regularly before injury. At the last follow-up, 9 (56.3%) had returned to their sports activities. Irrespective of the fracture type, less than 40% of the subjects were able to run, with no differences among the 4 groups. In all groups, the majority of the patients were able to walk barefoot with no differences; only in 8 cases (28.6%), the subjects complained about significant pain or difficulty walking barefoot. With regards to hindfoot inversion and eversion mobility, we found stiffness from absent to mild in 16 cases (57.1%), while moderate to severe stiffness was present in 12 cases (42.9%). In relation to these aspects, no significant differences were found upon statistical analysis (*p* = 0.23) comparing the *simplex fracture group* to the *complex group*. A total of 11 cases (39.3%) reported that it was impossible to wear the same shoes used before the trauma, or there were some restrictions on usable shoe shape. This condition was significantly higher (*p* = 0.01) in subjects of the *talar neck fracture group* with respect to the *body group.* Finally, correlating trauma-to-surgery interval with long-term clinical functional outcomes, a decrease of the scores (AOFAS, MFS, FFI-17 and SF-36 PCS) with increasing days of waiting was noticed, although statistical analysis did not show any correlation (*p* > 0.05) between timing of fixation and long-term clinical outcomes, even by correlating the scores reporting the best (AOFAS, *p* = 0.811) and worst results (FFI-17, *p* = 0.488) of our cohort (Fig.4).

### Complications

Among the 28 fractures treated, 10 (35.7%) presented early complications, with no significant differences between the *talar neck fracture* and the *body fracture groups* (*p* > 0.05). There were 6 (21.4%) malunions, 4 (14.3%) wound dehiscence, 2 (7.1%) cutaneous necroses and a single (3.6%) wound infection. On the other hand, late complications were much more frequent, such as PTA and AVN, affecting respectively 22 (81.4%) and 7 patients (25.9%). Among these last, one (3.7%) suffered only AVN, while the other 6 patients (22.2%) developed both AVN and PTA. The subtalar joint (STJ) was the most frequently affected (14 cases; 51.8%); followed by tibiotalar joint (TTJ) and talonavicular joint (TNJ) (10 cases, 37%; and 9 cases, 33% respectively). Further, most patients (7; 25.9%) presented more than one peritalar joint involved. Statistical analysis did not demonstrate significance in the differences between the *talar neck fracture group* and the *talar body fracture group*. In all 7 cases of AVN, revascularization resumed, avoiding the risk of dome collapse and the need for salvage procedures.

## Discussion

In this study 27 patients were treated by ORIF for 28 isolated, displaced, closed talar neck or body fractures. From the analysis of their A/P X-ray images after an interval of 6–8 weeks from the trauma, the typical sign of Hawkins was observed in 9 cases. However, the absence of this sign did not prevent satisfactory results from being achieved. Twelve cases with a negative Hawkins sign did not develop AVN, while the 7 remaining cases without this sign developed osteonecrosis. Hence, as confirmed by the literature [4, 35–37], the Hawkins sign has high sensitivity, but limited specificity.

The malunion rate after talar neck and body fractures has been reported in the range of 20–37% and 5–25%, respectively [2, 4, 15, 24, 36, 38, 39]. Given that, during the patient follow-up, no CT exam was performed and that most of the X-ray images were not taken with weight-bearing patients, our malunion rate, not statistically significant between the two fractures groups, was probably underestimated. Despite our efforts to prevent improper varus, it was not possible to avoid this most common deformity when medial comminution of the talus was associated. This is due to the fact that an AL exposure only permits visualization and a direct cortical reduction of the lateral talar neck. On the other hand, when an AM approach was performed, because of the inability to visualize the lateral aspect of the talar neck, it was impossible to judge the quality of reduction [15]. Consequently, our radiographic results and their correlation with the clinical outcomes are to be attributed to the use of only one surgical approach, instead of a dual incision approach, which would have minimized the probability of malunion [40]. Nevertheless, none of our patients have undergone subsequent corrective hindfoot osteotomy or arthrodesis. Nonunion is rare after talar neck fractures, occurring in 3–5% of cases [5, 9, 41], except after Hawkins type III (12%), mostly due to inadequate reduction [18]. However, in our cohort, no case of nonunion was recorded, as all fractures reached radiographic consolidation.

As in most early series, as well as several recent ones [4, 10, 15, 38, 42], internal fixation by screws was the predominant method of osteosynthesis during the period in which our patients were operated. Although some biomechanical studies have demonstrated no difference between screw-only fixation and combination screw-plate constructs [43, 44], some authors object that screw fixation can induce excessive compression in cases of a comminuted talar neck fracture [45]. On the contrary, plate fixation has the advantage of providing a more precise reduction, bridging areas of significant comminution, permitting talar length restoration and avoiding varus reduction errors [10, 46]. For these reasons, combination screw-plate construction of talar neck fractures has become the standard method of fixation [9, 20, 24, 39]. However, Beltran et al. do not advise its application on the medial neck as it could compress the tenuous vascular supply, or make superficial revascularization more difficult in the setting of AVN [47].

As suggested by some reports [6, 12, 13], a slightly aggressive single approach was adopted for this series as a strategy to avoid wound dehiscence or infection and cutaneous necrosis. Nevertheless, these early complications, occurred in 25% of our cases, reflecting the highest percentage reported by other studies (5–25%) [5, 6, 10, 11]. Regarding late complications, PTA incidence in the literature ranges from 50 to 100% increasing in the few studies with a longer follow-up [2, 4–6, 11, 36]. In our analysis, overall incidence of PTA was 81.4%. On the other hand, AVN was found in 25.9% of our series, in agreement with several studies [2, 4–6, 28, 36]. Moreover, no collapse of the talus dome was observed in any of the patients with AVN. In contrast, some authors have reported a ratio of dome collapse of up to 71.4% after AVN [5, 10], which would have required salvage surgery, such as triple arthrodesis [48] or total ankle prostheses [2].

For each questionnaire in our sample, the AOFAS score was better than values reported by other authors [4, 13, 38], while the MFS score was comparable to that recorded by Elgafy [10]; and the FFI-17 score was similar to that obtained by Vallier [5]. With regard to the quality of life, the SF-36 PCS questionnaire was higher than the reference value of the healthy population, in agreement with Xue’s results [9], but much better than those reported by Beltran [47]. Hence, these results mean that talar fractures do not affect health in a considerable way. In fact, we observed that in some cases, even non-optimal radiographic and functional outcomes were subjectively judged satisfactory by the patients using the VAS scale, so that more than 70% of our patients were completely satisfied.

Further, if we compare the *neck fracture group* to the *body group*, the AOFAS score of neck fractures was lower than the average of those of body fractures; however, without statistically significant difference, which is in accordance with other published studies [4, 13, 36]. Hence, fracture site does not seem to be a prognostic factor in limb functional recovery and remaining disability, though future prospective randomized clinical trials are certainly needed to confirm it. After comparing the two fracture patterns identified (the *simplex fracture group* and the *complex fracture group*), a considerable gap in terms of evaluation scores emerged. Statistical analysis found a significant difference in AOFAS, MFS, FFI-17 and SF-36 PCS scores. It is clear that talar fracture outcomes are directly correlated to trauma severity and fractures comminution, as reported by Sneppen [29].

Historically, neck fractures have often been treated urgently to reduce the risk of body AVN [11, 27, 49, 50]. However, in agreement with the recent literature [4, 5, 11], in our series, considering a surgical timing interval of 0–11 days (mean 3), delayed surgery had no impact on the development of AVN. In fact, although the clinical-functional scores tend to decrease with the number of days waiting for surgery, in agreement with the recent literature [4, 5, 11, 18, 51], we did not find any statistical correlation between timing of fixation and long-term clinical outcomes. Halvorson and Winter, in their recent review [2], were unable to identify any studies whose results would support emergent intervention for talar neck fractures. However, they underlined the difficulty in changing the common attitude of surgeons that talar fractures require emergent surgical treatment. On the contrary, when internal fixation cannot be safely undertaken on an urgent basis because of severe soft-tissue damage and swelling of the foot and ankle, the surgical delay allows a decreased rate of wound complications and infection, similar to that described for tibia pilon [52] and calcaneus fractures [53, 54].

Several potential limitations may influence the results of our study, such as its retrospective nature and the consequent lack of randomization. Further, radiographs analysed in our study were taken for clinical follow-up without a regularly prescribed basis and not for research purposes in a strict standardized manner. Hence, this aspect may have affected the different projections and altered the radiographic measures, particularly in relation to fracture classification and reduction quality. We are also aware that a single surgical approach and the method of fixation used are not representative of standard contemporary treatment for these rare injuries, even if both are still used for simple talar fractures. However, it must be considered, firstly, that the surgical procedures evaluated in this study were performed between 2007 and 2012, when, especially during the first years of this period, the most used osteosynthesis technique provided for screws. Secondly, to ensure that our initial experience with plate fixation, mostly used for non-isolated fractures, would not negatively influence the patients’ outcomes, we did not include the related operations in this study protocol. However, to the best of our knowledge, our report differs considerably from those previously published, which are often multi-centred, most of them relating only to neck fractures and using a limited number of clinical scores, and few reporting long-term outcomes of both isolated neck and body fractures. Another strength of this study is the good quality of data in our hospital database, collected by two independent investigators and recorded according to our standard aftercare algorithm. The analysis of the clinical and radiographic outcomes was carried out separately by two other researchers, and finally analysed by an independent statistician, blinded to the type of injuries in order to reduce bias.

## Conclusions

Based on the long-term radiographic and clinical-functional outcomes of the present study, it is possible to conclude the following:

- despite a high rate of long-term complications found in our series, satisfactory clinical results and even good quality of life were achieved;
- talar fracture location did not influence the final outcome: *complex fractures* characterized by comminution or important fragment displacement obtained lower clinical-functional scores compared to *simple fractures*, as expected.
- the Hawkins sign was confirmed as a positive prognostic factor with high sensibility;
- since operation timing did not influence AVN development and the achievement of good clinical results, these injuries do not require emergent surgical management by open reduction and internal fixation.

## Data Availability

The dataset supporting the conclusions of this article is available at our institution contacting the corresponding author.

## Abbreviations

A/P: Anteroposterior
AL: Anterolateral
AM: Anteromedial
AOFAS: American Orthopaedic Foot and Ankle Society
ASA: American Society of Anaesthesiologists
AVN: Avascular Necrosis
BMI: Body Mass Index
CT: Computed tomography
FFI-17: 17-Foot Functional Index
L/L: Lateral
MCS: Mental Component Summaries
MFS: Maryland Foot Score
ORIF: Open Reduction Internal Fixation
PCS: Physical Component Summaries
PTA: Post-traumatic Arthritis
ROM: Range Of Motion
SF-36: Short Form 36
VAS: Visual Analogue Score
